# Implementing neuropsychological rehabilitation following severe traumatic brain injury in a low-to-middle income country: a case report

**DOI:** 10.3389/fresc.2024.1393302

**Published:** 2024-06-12

**Authors:** Alexa Caitlin Soule, Taryn Jane Fish, Jill Winegardner, Leigh Schrieff-Brown

**Affiliations:** ^1^ ACSENT Laboratory, Department of Psychology, University of Cape Town, Cape Town, South Africa; ^2^ Department of Neurology, University Hospitals Cleveland Medical Center, Cleveland, OH, United States

**Keywords:** executive function, memory, neuropsychology, rehabilitation, single-case study, TBI

## Abstract

**Introduction:**

TBI incidence and distribution are largely overrepresented in low- to middle-income countries (LMICs), such as South Africa (SA), with substantial associated human and financial costs. However, access to rehabilitation for the public is severely limited and not standard practice in SA. Given this background, studies demonstrating the successful implementation of neuropsychological rehabilitation in a LMIC setting are important. Published studies of this nature are generally lacking in this context. Further, there is a need to evaluate interventions that can be implemented at a low cost. To this end, we report on a neuropsychological rehabilitation program for an individual with severe TBI in a LMIC context, aimed at improving his capacity for activities of daily living.

**Method:**

A 33-year-old, South African male who sustained a severe traumatic brain injury (TBI) partook in a neuropsychological intervention aimed at remediating functional deficits and enhancing independent functioning. The intervention utilised principles of Goal Management Training and external memory aids, with reliance on procedural memory and errorless learning, to target the participant's impairments in executive functioning and memory through the use of assistive technology—namely smart device applications.

**Results:**

Data collected pre- and post-intervention on formal neuropsychological measures demonstrated no significant change in cognition. However, observational data and qualitative feedback from the participant's family indicated notable improvement in performance on everyday tasks with reduced number of errors and reduced need for external prompting whilst completing intervention tasks across sessions.

**Discussion:**

In the context of severe TBI, neuropsychological rehabilitation can facilitate gains in independent functioning. This study provides support for the value of neurorehabilitation especially for interventions that can be rolled out at low cost and should serve as impetus for further such research in South Africa, where neuropsychological rehabilitation infrastructure and services are lacking.

## 1 Introduction

Whilst a global issue, TBI incidence and distribution are largely overrepresented in low- to middle-income countries (LMICs), such as South Africa (SA), with prevalence rates being three times higher in proportion to high-income countries (HICs) (1). Context-specific factors contribute to the increased burden of TBI in some LMICs (2). For example, in SA, TBI primarily results from high rates of interpersonal violence and road traffic accidents (3). Ironically, it is also within such countries, with higher rates of TBI, in which provision and access to neuropsychological rehabilitation is most limited (4, 5).

Access to rehabilitation for the general public is severely limited and not standard practice in SA, with unprepared and untrained families often left to cope with management of survivors of brain injury (5, 6). Additionally, there is huge economic burden associated with such injuries, with a recent estimate of costs associated with the management of TBI annually in South Africa being 60 million ZAR^1^ (7). Hence, the implementation of interventions to prevent and manage TBI are warranted in terms of both human and economic costs.

Given the lack of infrastructure for neuropsychological rehabilitation in SA, especially in the public sector, and the paucity of literature on SA-specific low-income intervention strategies, there is a need to evaluate and roll out affordable interventions (5). Thus, we present a summary of our efforts in executing a rehabilitation program for an adult male post-severe-TBI in Cape Town, South Africa. A review of the literature supports Goal Management Training (GMT), external memory aids, reliance on procedural memory and errorless learning as prominent strategies for ameliorating deficits of executive functioning and memory, which are frequently impaired following TBI (8, 9). Our research contributes towards the currently limited field of neuropsychological rehabilitation in SA.

## 2 Case description

The case participant is a 33-year-old male (referred to as FS) who sustained a severe TBI (Glasgow Coma Scale score of eight on site and five upon hospital admission) following a motor vehicle accident (MVA) in November 2016. FS was referred to one of the researchers for neuropsychological rehabilitation from a local hospital. His first language is Afrikaans^2^, but he is also fluent in English. His highest level of education is grade 12 (i.e., completed high school). Notably, most of his adult life was spent as a professional athlete in a contact sport. FS retired from this a few years prior to the accident and had started a new job in packaging sales at the time of the accident.

FS' neuropsychological reports indicated TBI with diffuse axonal injury, which resulted in severe executive dysfunction and memory impairments. FS' full-scale IQ is markedly low (67), with his verbal IQ (78) markedly higher than his performance IQ outcome (60). Cognitive assessment revealed deficits in memory, attention, and executive functioning. FS' dysexecutive syndrome was characterized by deficits in attention, planning, strategising, inhibition, processing speed and problem solving. Regarding his memory, FS had both encoding and retrieval deficits. While FS' explicit memory systems were impaired, his implicit memory appeared relatively preserved. In terms of physical functioning, FS sustained a talus ankle fracture during the MVA which resulted in mild difficulties walking. However, no sensory impairments or pain were reported by FS and his family. He remained dependent on caregivers to accomplish activities of daily living and was unfit for employment.

Lack of insight is common following TBI which makes obtaining informed consent from participants with TBI an ethical challenge (10). As such, common practice is to request consent from the next-of-kin (11). We requested written consent from FS' fiancé for his participation in the study and asked FS to give written assent. Additionally, at each session, verbal assent was sought from FS. We obtained ethical clearance for this study from the University of Cape Town Psychology Department's Research Ethics Committee—reference number PSY2019-018.

## 3 Methods

### 3.1 Intervention overview

**Figure 1:**
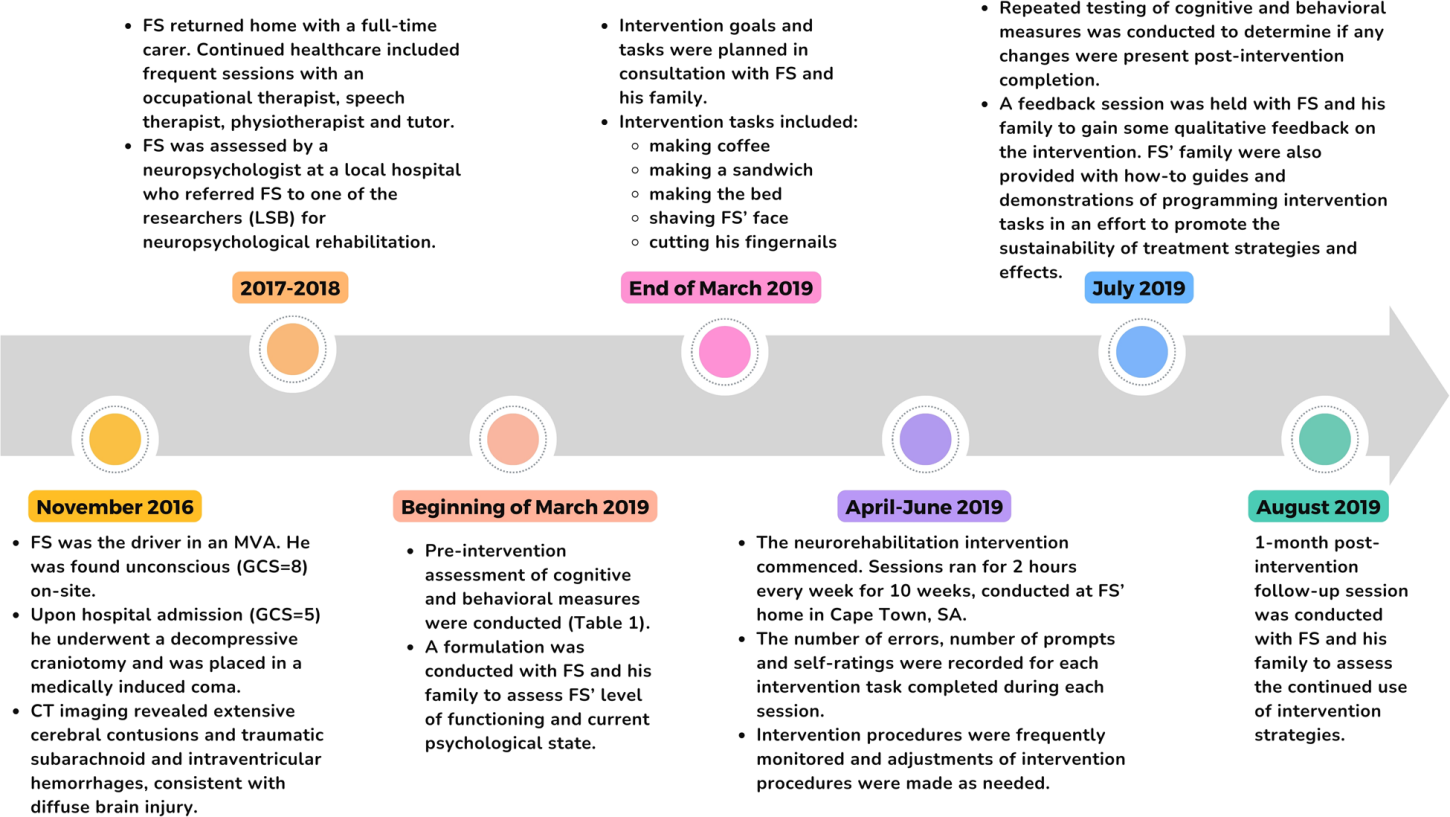
A brief overview of participant progression from the time of the MVA up until 1-month post-intervention follow-up (developed in accordance with CARE guidelines).

Figure 1 displays a timeline of events.

### 3.2 Intervention design

The intervention was carried out by a senior neuropsychologist (LSB) and two honors students (ACS and TJF) at the University of Cape Town. We met with FS weekly at his home for about 2 h per week over 10 weeks, with a follow-up session one-month post-intervention to assess the continued use of intervention strategies. All assessments and the intervention itself were conducted at the participant's home for his convenience, to reduce testing anxiety, and to increase the ecological validity of the intervention. The intervention strategies were developed in accordance with recommendations made in The Brain Injury Rehabilitation Workbook (12). Pre-intervention cognitive assessment provided insight into FS' cognitive strengths and impairments. Next, a formulation was conducted to summarize potential factors influencing FS' level of functioning and current psychological state, gathered through cognitive and behavioural measures (described below), and via discussion with FS, his fiancé, parents, and caregiver.

Based on his cognitive profile and formulation, executive functioning and memory were identified as target areas of the intervention and subsequent intervention tasks were chosen in consultation with FS and his fiancé. Psychoeducation was given to explain the mechanisms of FS' injury, outcomes, and treatment options, thereby enhancing the family's insight into FS' condition (12). Research suggests that psychoeducation is effective in improving family functioning and adjustment to TBI, while also reducing distress and burden of care (13, 14).

Upon completion of the intervention, a step-by-step guide with instructions on how to program new tasks was given to FS’ fiancé to ensure continuity and sustainability of the intervention beyond the structured sessions (see Supplementary Material S1). It aimed to empower FS' support network with the tools and knowledge necessary to reinforce and maintain the strategies implemented during the intervention, promoting long-term independence and success in managing daily tasks and routines.

### 3.3 Intervention tasks

Intervention tasks comprised five routine daily tasks with which FS and his fiancé indicated they would like assistance—namely, making coffee, making a sandwich, making the bed, shaving FS' face, and cutting his fingernails. As FS became proficient with these tasks, additional activities like brushing hair and making tea were introduced in subsequent weeks. The tasks varied week to week based on necessity (e.g., whether shaving or nail cutting was needed) and FS' preference. Decreased self-awareness is a common outcome following TBI (12, 15), hindering rehabilitation progress due to unrealistic goal-setting and reduced motivation (13). Research suggests that improving participants' awareness of their impairments can thus optimize gains from rehabilitation (12, 16, 17). To achieve this, we had FS rate his performance on intervention tasks on a five-point scale—with higher scores reflecting better execution. This aimed to promote self-reflection and awareness. We also provided our own ratings, highlighting any discrepancies to improve FS' insight.

#### 3.3.1 Checklists

**Figure 2:**
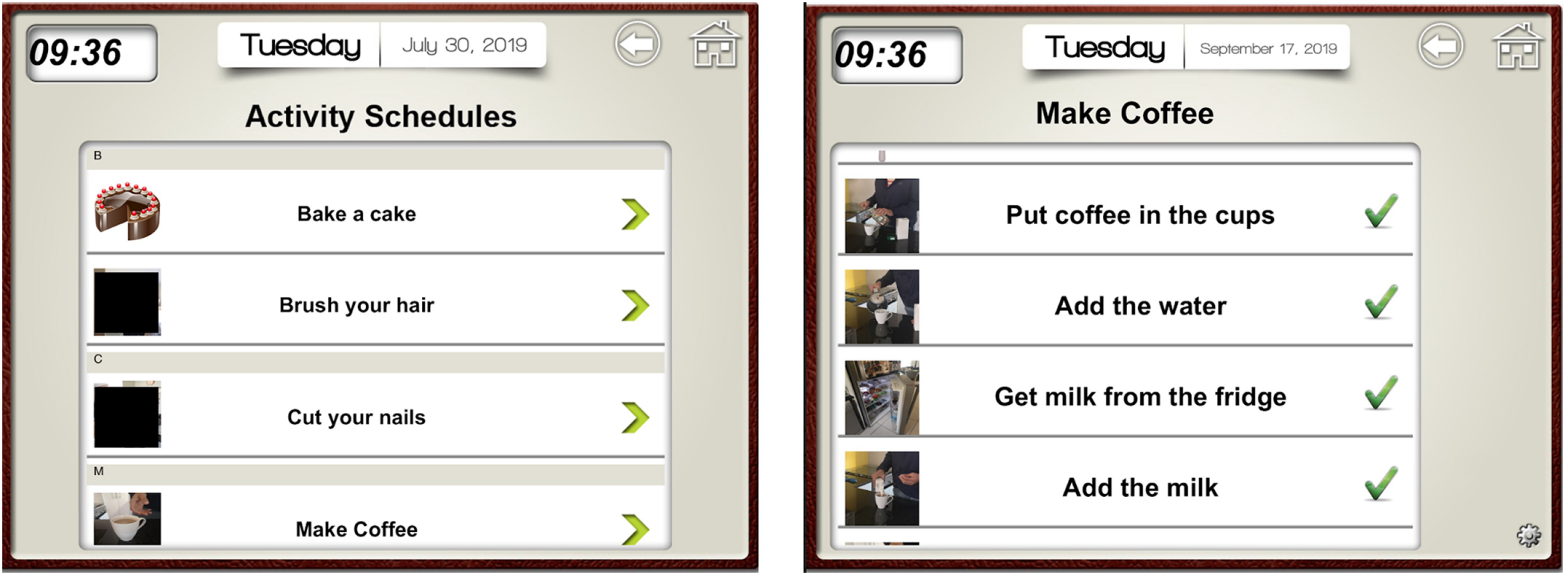
Screenshot from visual schedule planner iPad application. The image on the left depicts the list of tasks that FS could choose from. The image on the right depicts an example of checklist steps for the task of making coffee. Specific photographs from FS environment were inputted next to each step as a visual cue to aid in memory retrieval of the object/location required for each step. Tick marks in the image denote that a task has been completed successfully.

Applying the principles of GMT, we constructed a checklist of steps for each intervention task. We broke each task down into explicit and manageable steps. These were programmed into the Visual Schedule Planner application on FS' iPad featuring step-by-step instructions and custom images (e.g., photos of items and locations in FS' home environment; see Figure 2). This addressed FS' memory difficulties as it provided a prompt for where to find necessary items. Initially, we observed FS performing tasks independently to assess his proficiency. If his existing approach was effective, we aligned our checklists with his natural sequence of ordering steps. We introduced the checklists in session two and supervised FS using these checklists in subsequent sessions, offering prompts if needed to reduce the chances of errors occurring [i.e., errorless learning (18)]. After each session, we reviewed the checklists for each task, altering steps which proved difficult or confusing for FS. For example, we added a step to ask for help if the milk had run out.

#### 3.3.2 Memory aid

To target FS' memory impairments, we programmed alert notifications into the Visual Schedule Planner application on his iPad. These notifications served as reminders to perform intervention tasks at specific times during the day—when the alert sounded. However, the default notification sounds were insufficient, so we switched to using Google Calendar for louder alerts, starting from session seven. FS practiced responding to these notifications during intervention sessions. Additionally, we collaborated with FS' fiancé to set up reminders on Google Calendar for tasks beyond the intervention, like medication reminders, starting from session 10.

### 3.4 Outcome measures

Assessment of intervention success was measured by: (1) a combination of cognitive and behavioural measures (see Table 1) with FS and his family before the intervention, and at the end of the intervention (approximately three months post-initial assessment), (2) within-intervention assessment of intervention tasks recording the number of errors, the number of prompts and self-ratings (as described above), and (3) qualitative feedback from FS and his family (see Supplementary Material S2).

**Table 1:** Summary of cognitive and behavioral measures utilized pre- and post-intervention to, in part, determine intervention efficacy.

Cognitive measures and subtests	Description of domains targeted
Wechsler adult intelligence scale—third edition (WAIS-III) (19)	General intellectual functioning (IQ)
Digit span	Attention and working memory
Symbol search	Processing speed
Digit symbol coding	Processing speed
Wechsler abbreviated scale of Intelligence—second edition (WASI-II) (20)^a^	General intellectual functioning (IQ)
Vocabulary	Knowledge of word definitions, verbal concept formation and crystallized intelligence
Similarities	Abstract reasoning and understanding the relationships between words and concepts
Block design	Visuospatial functioning, motor functioning and problem solving
Matrix reasoning	Non-verbal abstract reasoning and perceptual organization
Delis-Kaplan executive function system (D-KEFS) (21)	Executive functioning
Tower test	Problem solving, spatial planning abilities, and ability to follow instructions and learn rules
Letter and category fluency	Verbal generativity and cognitive flexibility
Wide range assessment of memory and learning—second edition (WRAML-2) (22)	Learning and memory: immediate recall, delayed recall and recognition
Behavioral measures	Description of domains assessed
Patient-reported outcome measurement information system—29 version 2.0 (PROMIS-29) (23)	General health of the participant, across seven health domains (pain interference, depression, anxiety, physical functioning, fatigue, sleep quality and social activities)
Dysexecutive questionnaire—revised version (DEX-R) (24)	The impact of dysexecutive syndrome on daily living
Self-concept questionnaire (SCQ) (25)	Overall affect and self-esteem
Quality of life after brain injury (QOLIBRI) (26)	General quality of life after sustaining TBI, including cognition, emotions, daily and physical functioning, personal and social life
Caregiver strain index (CSI) (27)	Areas of concern or challenges, experienced by family and caregivers, caused by the patient's care demands
Patient competency rating scale (PCRS) (28)	Awareness of deficits

^a^ The WASI-II, which measures IQ and is unlikely to change significantly over time, was only administered pre-intervention and not post-intervention.

#### 3.4.1 Within-intervention assessments

After obtaining FS' verbal consent, we video recorded him performing the intervention tasks in every session. We analyzed these recordings retrospectively, noting errors (such as deviating from the checklist or incomplete steps) and prompts (actions or verbal instructions given to refocus FS' attention or correct errors). Our own execution ratings, based on a five-point scale described above, gauged task mastery. The difference (formula: Difference = Our rating—FS' rating) between our and FS' ratings gauged his awareness. New tasks were introduced once previous tasks earned consecutive perfect execution scores (i.e., 5/5) over two sessions. Mastered tasks were then either dropped or were still repeated as part of FS' routine. We qualitatively assessed FS' response to Google Calendar alerts on his iPad.

#### 3.4.2 Qualitative feedback

Following the intervention program's conclusion, we arranged a feedback session with FS and his family to gather qualitative evaluations. Prior to the session, all members were asked to complete open-ended feedback forms regarding their experiences, any observed changes, concerns, and suggestions (see Supplementary Material S2). During the session, we provided an overview of the intervention process, explaining the strategies employed to address FS' memory and executive functioning challenges, as well as the use of the iPad application. Attendees were encouraged to ask questions and share comments during the session.

#### 3.4.3 Statistical analysis

To assess whether the change in cognitive and behavioural scores from pre- to post-intervention testing was statistically significant, we used the Reliable Change Index (RCI). Differences at the 68.26%, 95% and 99% confidence interval are recorded with change at the 95% confidence interval being considered clinically significant (29). This outcome was calculated using a reliable change generator, using the following RCI formula:

SEd=√2p(Se)2,whereSe=s(√1−rxx)

Where *s* stands for the standard deviation and rxx stands for the test-retest reliability coefficient (29).

## 4 Findings

### 4.1 Cognitive and behavioral measures

FS showed consistently low scores on all cognitive measures (within the extremely low range), with no clinically significant changes post-intervention according to RCI analysis. Regarding behavioral measures, there was no significant change for FS on the DEX-R, QOLIBRI, and CSI. No notable changes were found in most PROMIS subtests completed by FS. However, there was a slight increase in anxiety and a decrease in fatigue, although statistically significant only at the 68.26% confidence interval. Ease of physical functioning decreased significantly, with a confidence interval of 95%, indicating greater difficulty in this domain. Further, FS demonstrated a significant change, at the 95% confidence interval, on the Robson SCQ, indicative of increased levels of self-esteem. On the PCRS (measure of awareness of deficits), only FS' fiancé showed significant positive change (at the 95% confidence interval).

### 4.2 Within-intervention assessments

**Figure 3:**
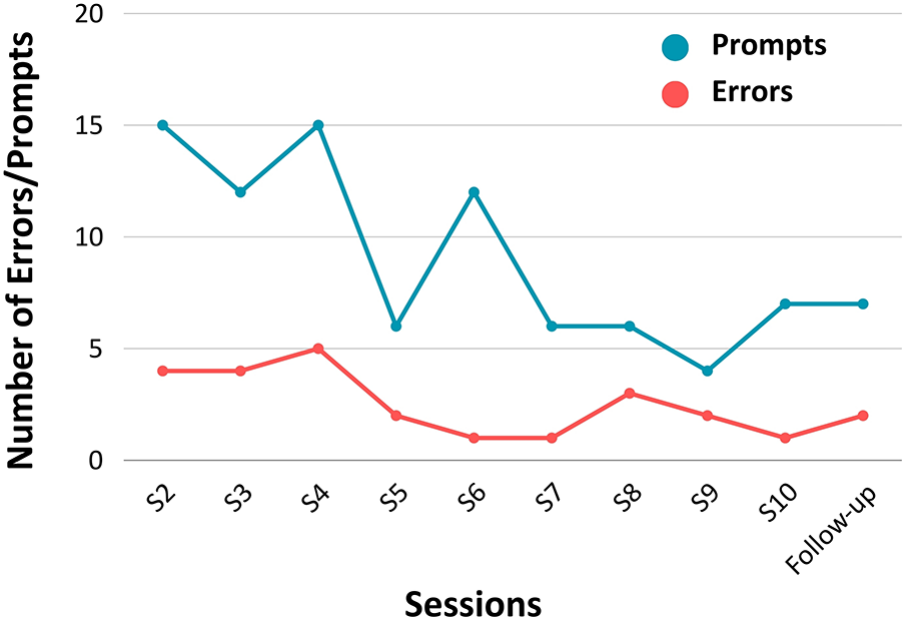
Average number of errors made and average number of prompts required by FS per task, per session (*N *= 1).

The average number of errors made per session across each task is recorded in Figure 3. On average, the number of errors decreased over time, extending beyond the intervention period. Notably, the number of errors made during the follow-up session for all activities was lower than FS' first completion of each task. Additionally, the nature of errors also evolved over time. Initially, errors involved retrieving incorrect items or searching in the wrong location (e.g., retrieving water instead of milk). However, as time progressed, FS referenced the checklist more diligently and errors shifted towards incomplete (but logical) actions (e.g., retrieving peanut butter but not bread when instructed to retrieve both). The average number of prompts given across tasks, per session, are recorded in Figure 3. Similar to the trend in error reduction, the average number of prompts required by FS decreased from session two to 10. At the follow-up session, the number of prompts per task was lower compared to FS' first completion of each task. This declining trend, depicted in Figure 3, thus held in the one month following the intervention period.

In terms of the difference in execution ratings between FS and us, on average in early sessions, there are more negative ratings, suggesting FS rated himself higher than us. This contrasts to later sessions where there are more positive ratings suggesting FS rated himself lower than us. Notably, this trend is held in the follow up session with FS one-month post-intervention.

The auditory alert notifications were introduced in session six using the Visual Schedule Planner application. Once we changed the alerts to Google Calendar in session seven, which provided much louder and commanding alerts, FS was noticeably more attentive. He began reading the notification aloud, before turning off the alarm and promptly proceeding to perform the task that it instructed by making use of the programed checklists.

### 4.3 Qualitative feedback

During the feedback session, FS provided limited input, describing the intervention as “good” and “professional” on the feedback form. However, his family and caregiver reported notable and meaningful improvements in daily functioning. FS had begun using the iPad to independently complete tasks (e.g., making the bed; preparing breakfast). Prompted by Google Calendar alerts, FS now performs tasks without hesitation. His parents highlighted his increased willingness to assist with household chores without being prompted, reducing frustration and conflict—as noted by FS' fiancé, “*He basically helps himself a lot more than before and this in turn helps me and [caregiver]. No more “fighting” to do a task”.* FS' fiancé had even added a new task (tying shoelaces) to the Visual Schedule Planner application.

## 5 Discussion

In this report, we detailed a neuropsychological rehabilitation program that made use of task checklists (based on GMT) and an external memory aid, mediated by errorless learning and reliance on procedural memory. While formal neuropsychological measures showed no significant change post-intervention, observational data and qualitative feedback indicated notable improvement in performance on tasks of daily living—suggesting the intervention was effective in its aim of increasing the participant's capacity for everyday functioning.

The lack of change noted on formal cognitive measures may be a function of injury severity, but it may also be related, in part, to the compensatory methods of remediation utilized. Both the checklists and Google Calendar aimed to bypass (rather than restore) FS' cognitive impairments. As such, the lack of change on the cognitive measures is relatively unsurprising (12, 30, 31). Further, given that our aim was not to change cognitive scores, but rather to improve tasks of daily living, this lack of change on formal cognitive measures does not detract from the intervention's efficacy (30). FS' behavioral measures also showed minimal change, which may be explained by his impairments (e.g., FS' profound memory impairments may have affected response accuracy on the PROMIS questionnaire, which requires recalling experiences from preceding days) (32). While FS' fiancé qualitatively described decreased caregiver strain, no such change was reflected on the CSI, which only measures either the absence or presence of caregiver strain, but does not capture varying levels of burden (33). Future studies can adopt a more appropriate method of evaluation by addressing what meaningful *functional* changes occur as a result of the intervention (17).

While errors decreased throughout intervention sessions, suggesting enhanced task completion, fluctuations are typical in participants with TBI (34), as shown in our data. Nevertheless, on average, FS made fewer errors over time, supporting the intervention's effectiveness in enhancing his ability to effectively perform tasks of daily living. Similarly, there was a decline in prompting needed by FS across sessions, which aligns with the errorless learning approach [where more prompting is provided initially to prevent incorrect learning (18)] and reflects his increased confidence and reduced need for assistance. The decrease in errors and prompting suggests FS increasingly relied on procedural memory for task completion. Repetition allowed him to consolidate procedural memories for each activity (35), reducing the need for executive functions like planning and sequencing. In this way, Google Calendar proved to be an effective external memory aid, strengthening the reinforcement between alert sound and task performance. This echoes recent research supporting the use of technology, such as smartphones, in TBI rehabilitation (36) [see e.g., Baldwin and Powell (37); McDonald et al. (38)]. Our findings support the use of implicit memory strategies to compensate for executive dysfunction and declarative memory impairment following severe TBI (39, 40).

While supervision remains important, intensive monitoring is now less necessary, thereby reducing caregiver strain and promoting FS' independence. These positive changes observed at one-month follow-up demonstrate the intervention's ecological validity and sustainability. FS' fiancé's addition of a new activity and continued use of checklists and reminders by the family highlights the ongoing usefulness of the intervention. Family involvement in practicing intervention tasks outside of the intervention sessions is essential in promoting intervention sustainability and generalizability (41). Such methods hold promise for delivering effective neuropsychological interventions in LMIC contexts, like South Africa, in which rehabilitation infrastructure is limited.

### 5.1 Limitations

Our 10-week intervention could be optimized by increasing the frequency of practice on intervention tasks, leveraging the benefits of procedural memory rehearsal (42). Lengthening the intervention duration or integrating our tasks and strategies into the sessions of other health professionals who work with FS weekly could achieve this. Additionally, while our intervention focused on compensatory methods, considering FS' distractibility, integrating restorative attentional training could have been beneficial (43). Future research should explore the feasibility of multimodal approaches in neuropsychological rehabilitation in LMICs. Lastly, in the current study, the researchers reviewed and coded the video recordings of FS' weekly task performance. To eliminate any possible bias, future research should employ independent researchers, blinded to the chronological order of sessions, to code and evaluate the recordings.

### 5.2 Conclusions

TBI, a leading cause of brain injury globally, poses significant challenges to cognitive, emotional, and psychological functioning (44). Our intervention's success in enhancing FS' ability to perform functional tasks highlights the importance of neuropsychological rehabilitation in addressing these impairments, even in severe TBI. Additionally, our research contributes to the currently limited body of research concerning neuropsychological rehabilitation within LMIC contexts. Despite the scarcity of rehabilitation services in such settings, our study demonstrates the feasibility and effectiveness of neurorehabilitation efforts, providing impetus for further research and interventions.

## Data Availability

The raw data supporting the conclusions of this article will be made available by the authors, without undue reservation.

## Footnotes

^1^ZAR stands for South African Rand, the official currency of South Africa.

^2^One of South Africa’s eleven official languages.
