# Supplementary material for: Implementing neuropsychological rehabilitation following severe traumatic brain injury in a low-to-middle income country: a case report
Source: Front Rehabil Sci. 2024 Jun 12;5:1393302. doi: 10.3389/fresc.2024.1393302 (PMC11199878; doi:10.3389/fresc.2024.1393302)
Supplement: Supplementary file 1 [file Datasheet1.pdf]

Supplemental Material 2 – Qualitative Feedback Template

Feedback form for FS

We have so enjoyed working with you these past few months. Thank you for your time and hard work. We would really appreciate any thoughts or feedback you might have. Could you please take some time to answer the following questions?

Overall, how would you describe the sessions with us?

Have you found our sessions helpful? How?

Have you noticed any changes in your daily life in your:

Routine:____

____

____

_

Emotions:____

____

____

Memory:____

____

____

Any other thoughts or comments, critiques or recommendations?

____

____

____

____

____

____

____

____

Feedback Form for Parents & Fiancé

Feedback

We have so enjoyed working with your family. Thank you for your time and cooperation. We would really appreciate any thoughts or feedback you might have. Could you please take some time to answer the following questions.

Name: ___________________________

Relation to [FS]: ________________

Overall, how did you find the experience?

Have you observed any noticeable changes in [FS]? Speech, behaviour, emotions or personality?

Have there been any meaningful changes in your daily routine or life in general?

Have these sessions raised any concerns or brought your attention to anything new?

Any other thoughts or comments, critiques or recommendations?

Feedback Form for Caregiver

Terugvoering

Dit was ‘n plesier om saam met u te werk. Dankie vir u tyd en samewerking. Ons sal dit waardeer as u enige idees of terugvoering vir ons het. Kan u asseblief hierdie vorm vir ons invul?

Naam: ____

Hoe het u hierdie ervaring ondervind?

____

____

____

____

____

Het u enige veranderinge in Shandre waargeneem in terme van spraak, emosies, gedrag of persoonlikheid?

____

____

____

____

____

____

____

Was daar enige noemenswaardige verandering in u daaglikse roetine of lewenskwaliteit oor die algemeen? Gee asb voorbeelde indien van toepassing.

____

____

____

____

____

Het hierdie sessies enige kommerwekkende kwessies aan die lig gebring of beklemtoon of dalk iets nuuts onder u aandag gebring?

Het u enige verdere kommentaar, kritiek of aanbevelings wat u met ons wil deel?
