# Supplementary material for: Implementing neuropsychological rehabilitation following severe traumatic brain injury in a low-to-middle income country: a case report
Source: Front Rehabil Sci. 2024 Jun 12;5:1393302. doi: 10.3389/fresc.2024.1393302 (PMC11199878; doi:10.3389/fresc.2024.1393302)

Supplementary Material 1

Visual Schedule Planner – How to Guide

**Please note that images and identifying information has been blacked out to protect the identity of FS.*

<u>VISUAL PLANNER: Homepage</u>

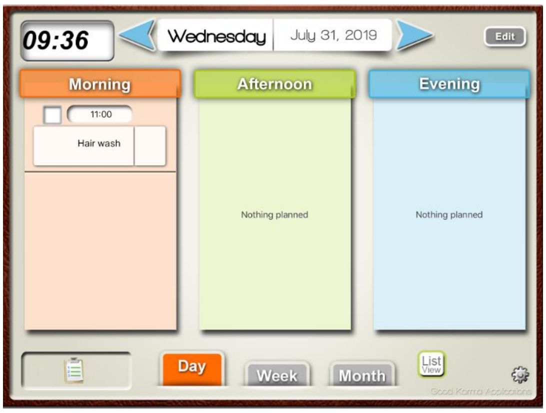

<u>DAY VIEW</u>

*You can ask [FS] to check the date and time using this app

<u>WEEK VIEW</u>

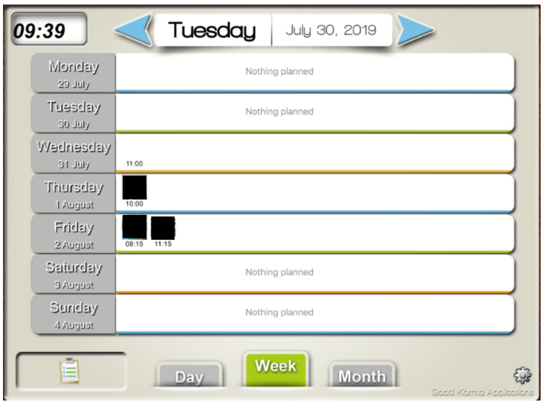

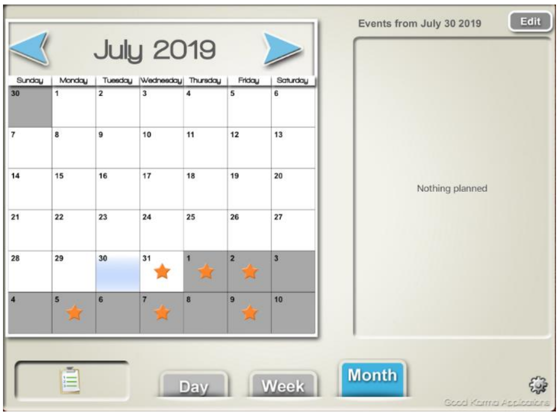

<u>MONTH VIEW</u>

*A star will show if there is an event/activity planned for that day

EDITING ON THE APP

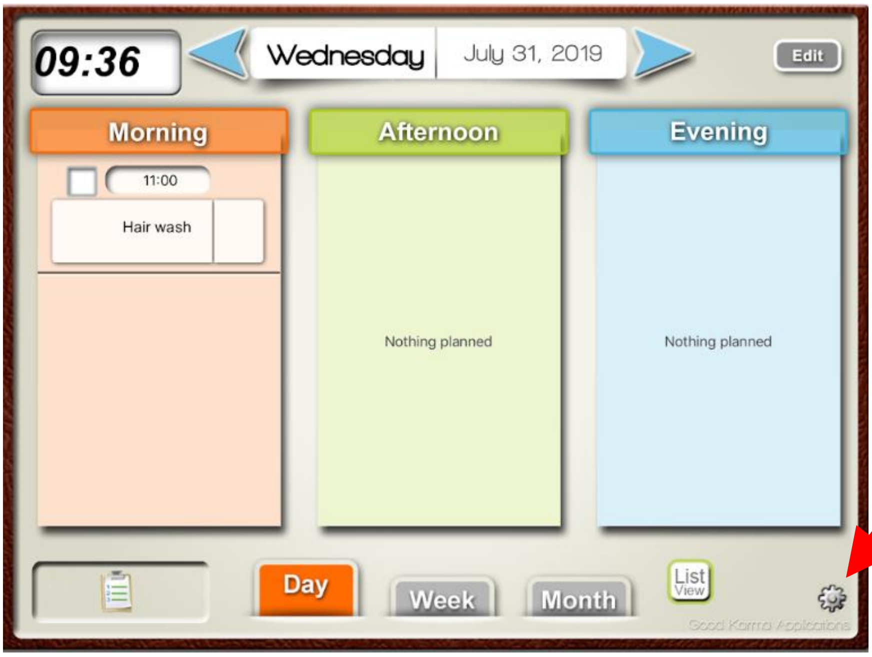

Step 1

Click on the settings button on the bottom right hand corner

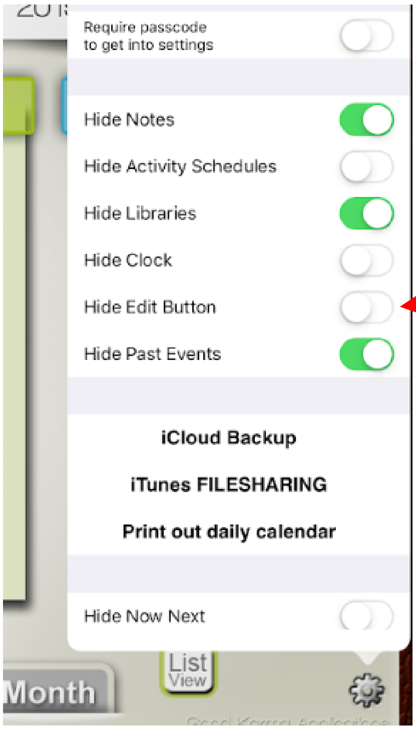

Step 2

Ensure that the "Hide Edit Button" is unselected

You will want to select this button before handing it back to [FS]

If you would like, you can set up a passcode at the top of the settings page

ACCESSING HIS ACTIVITIES

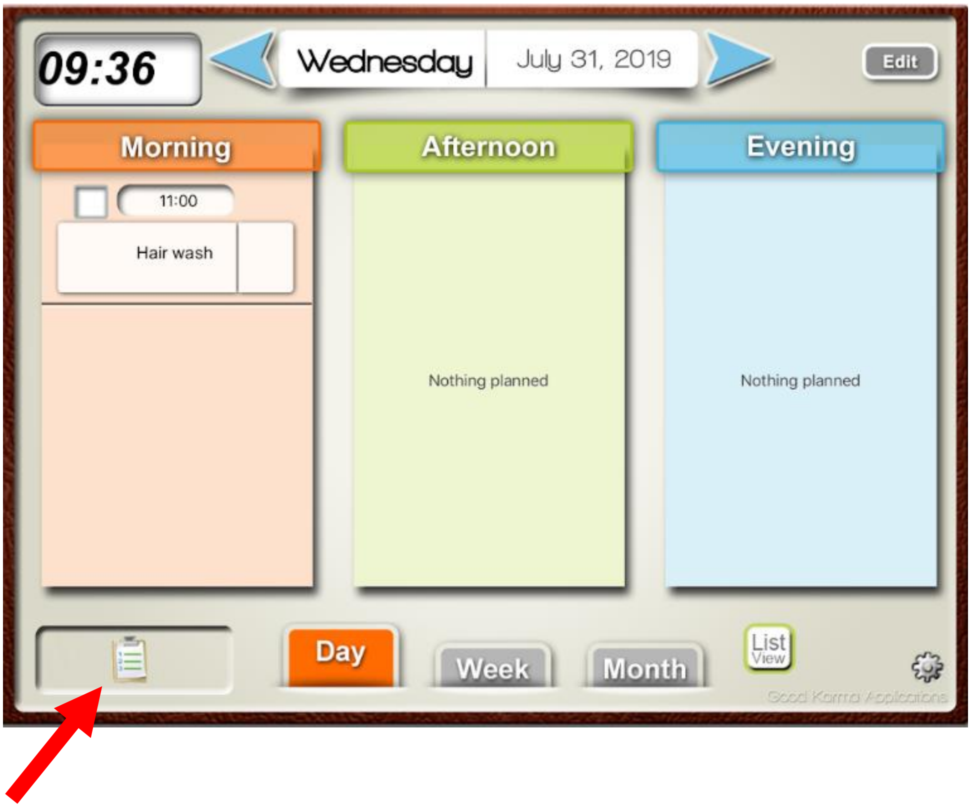

You can access a list of his activities using this button

You will see what is displayed below:

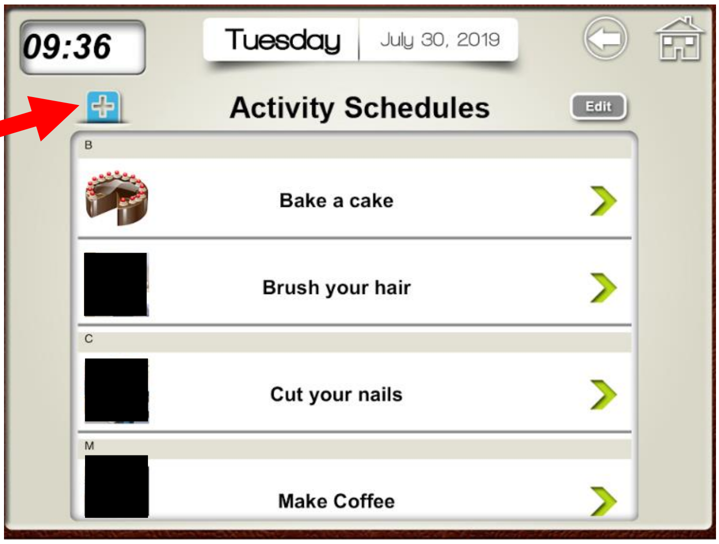

You can **add a new activity here**

You can return to the homepage using this button

<u>**ADDING A NEW ACTIVITY**</u>

Add a **picture** of the activity
(e.g. a cup of coffee that [FS] has made)

Add a new step(s) here

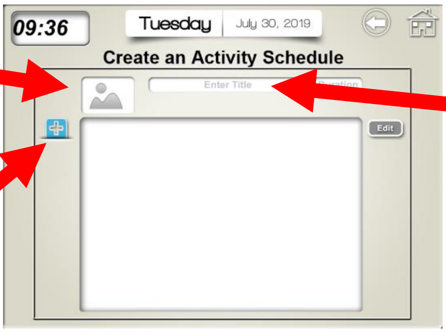

Add the **title** of the activity here

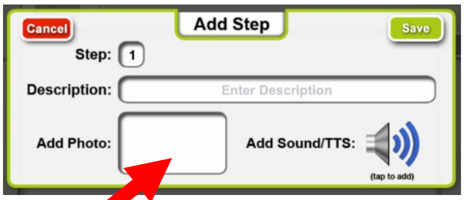

You can enter the title of the step here

Make it as descriptive as possible but notice that it won’t display if it’s more than 5 or 6 words

You can add a photo here

You can add an image by searching from an inbuilt list here (“Icon Library”)

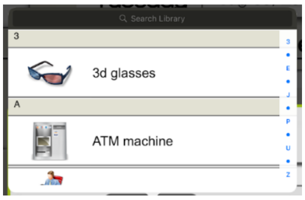

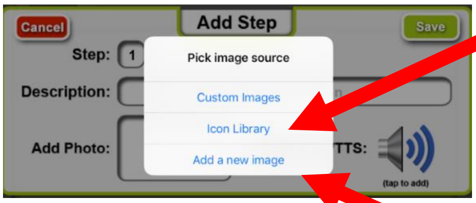

You can add an image from the internet or from your camera roll here (“Add a new image”)

It is better to take photos in your environment (e.g. of the kettle in your kitchen) as it will help prompt [FS] where to look if he is unsure

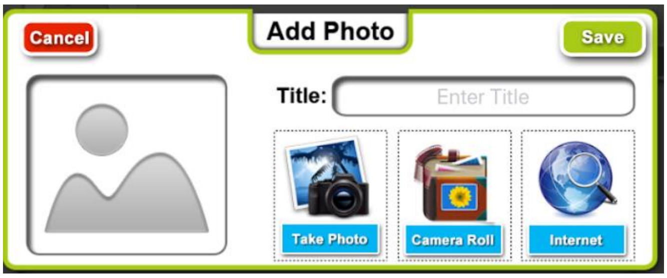

Supplement: Supplementary file 2 [file Datasheet2.pdf]
